# Supplementary material for: Antiviral Properties of Silver Nanoparticles against SARS-CoV-2: Effects of Surface Coating and Particle Size
Source: Nanomaterials (Basel). 2022 Mar 17;12(6):990. doi: 10.3390/nano12060990 (PMC8950764; doi:10.3390/nano12060990)
Supplement: Supplementary file 1 [file nanomaterials-12-00990-s001.zip › nanomaterials-1603195-supplementary.pdf]

nanomaterials

Supplementary Materials

Antiviral Properties of Silver Nanoparticles against SARS-CoV-2: Effects of Surface Coating and Particle Size

Qinghao He [1,†], Jing Lu [2,†], Nian Liu [3,†], Wenqing Lu [4], Yu Li [1], Chao Shang [2,*], Xiao Li [2,*] and Ligang Hu [1,3,5,*], Guibin Jiang [1,3]

[1] State Key Laboratory of Environmental Chemistry and Ecotoxicology, Research Center for Eco-Environmental Sciences, Chinese Academy of Sciences, Beijing 100085, China; qhe001@outlook.com (Q.H.); yuli_st@rcees.ac.cn (Y.L.); gbjiang@rcees.ac.cn (G.J.)
[2] Changchun Veterinary Research Institute, Chinese Academy of Agricultural Sciences, Changchun 130122, China; lujing0819@126.com
[3] School of Environment, Hangzhou Institute for Advanced Study, University of Chinese Academy of Sciences, Hangzhou 310024, China; liunian_goodluck@126.com
[4] School of Life Sciences, Hebei University, Baoding 071002, China; moonlewini@outlook.com
[5] School of Environment and Health, Jianghan University, Wuhan 430056, China
* Correspondence: shangchao1290@126.com (C.S.); skylee6226@163.com (X.L.); lghu@rcees.ac.cn (L.H.)
† These authors contribute to the study equally.

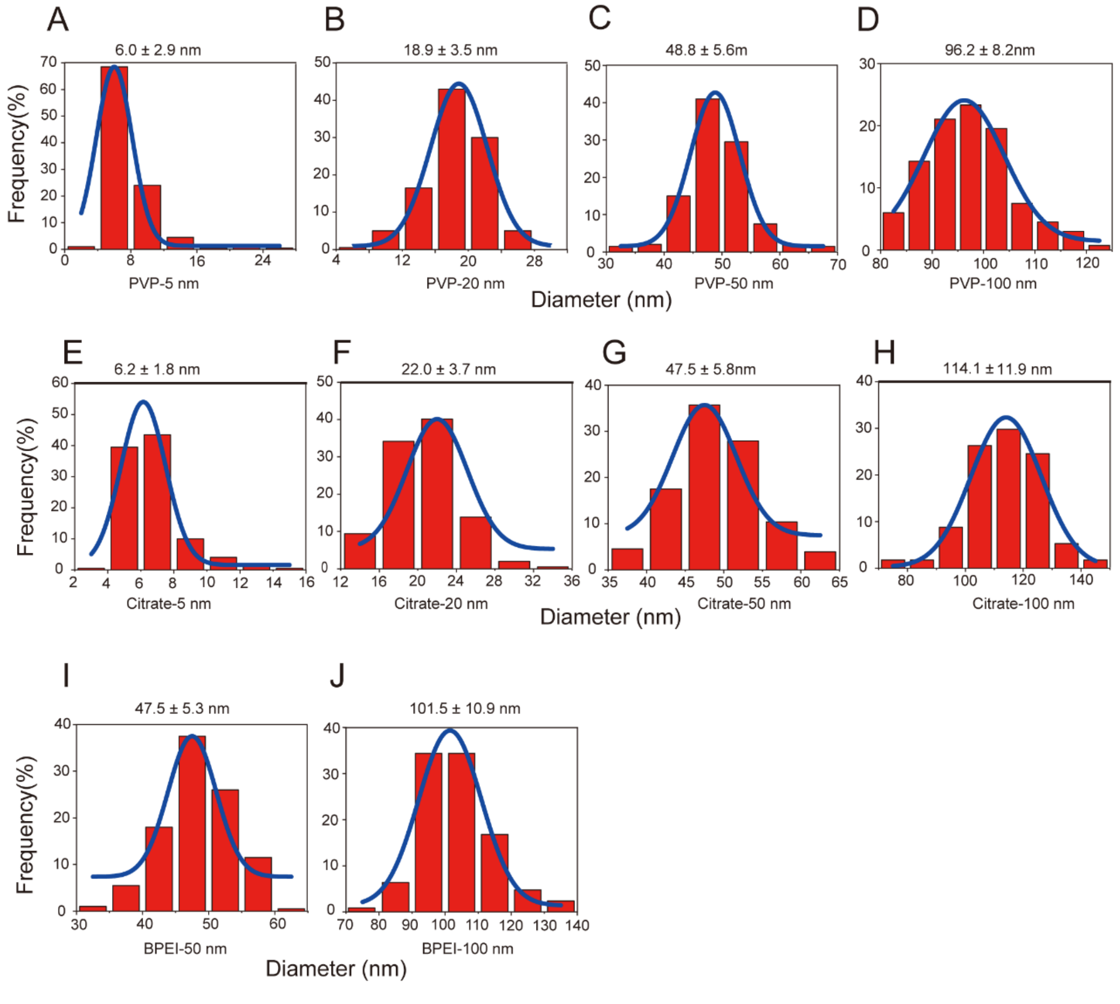

Figure S1. The size distributions of AgNPs with different particle sizes which modified by three materials in TEM images. Particle size distribution of PVP modified AgNPs with different particle sizes: 5 nm (**A**), 20 nm (**B**), 50 nm (**C**), 100 nm (**D**); (**E-H**) Particle size distribution of citrate-modified AgNPs: 5 nm (**E**), 20 nm (**F**), 50 nm (**G**), 100 nm (**H**); (**I,J**) Particle size distribution of BPEI modified AgNPs with different particle sizes: 50 nm (**I**), 100 nm (**J**).

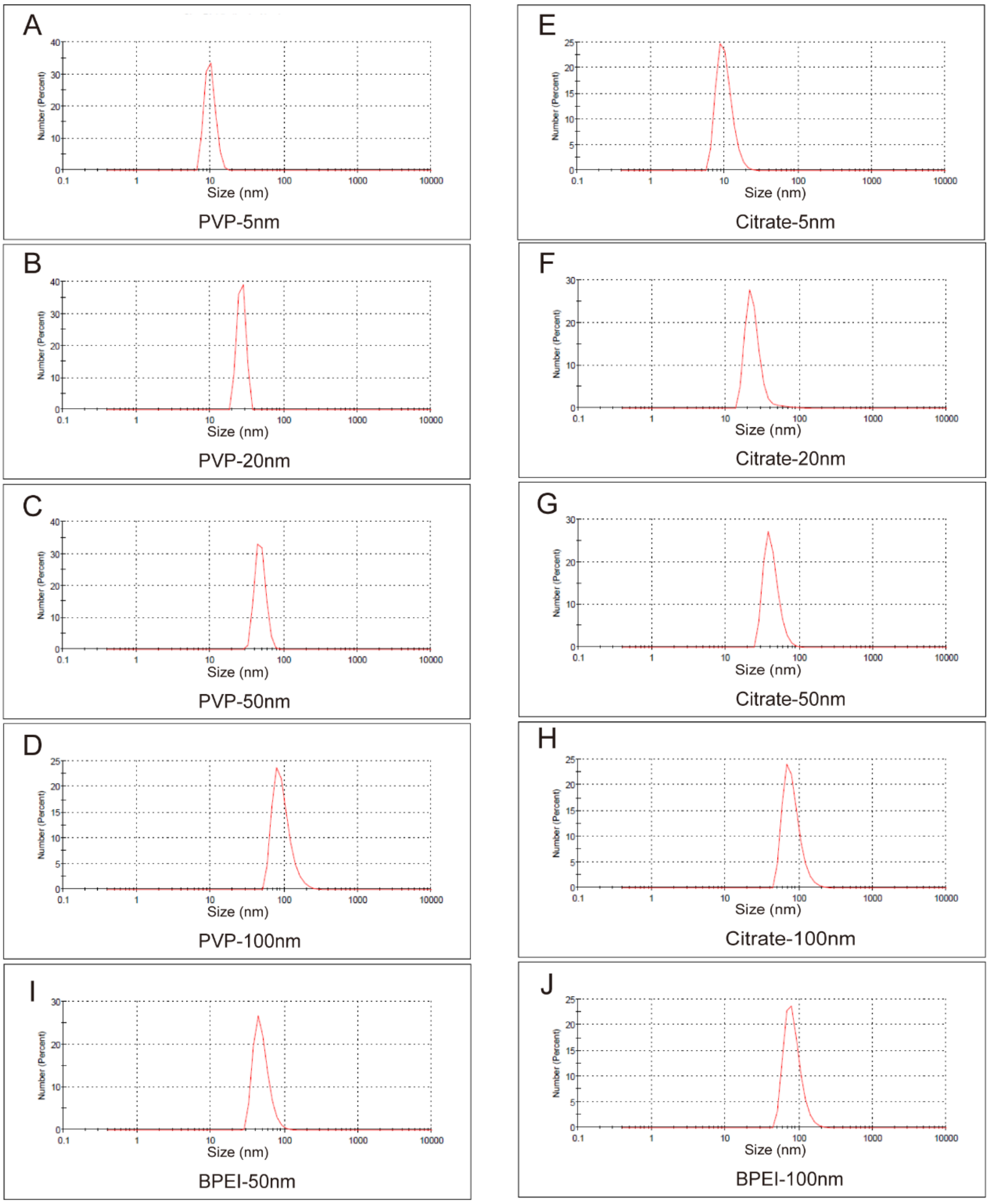

Figure S2. The hydrodynamic sizes distribution measured via dynamic light scattering. The hydrodynamic size distribution maps of PVP modified AgNPs with different particle sizes: 5 nm (**A**), 20 nm (**B**), 50nm (**C**), 100nm (**D**); (**E**–**H**) hydrodynamic size distribution of citrate-modified AgNPs: 5 nm (**E**), 20 nm (**F**), 50nm (**G**), 100nm (**H**); (**I**,**J**) hydrodynamic size distribution of BPEI modified AgNPs with different particle sizes: 50nm (**I**), 100nm (**J**).

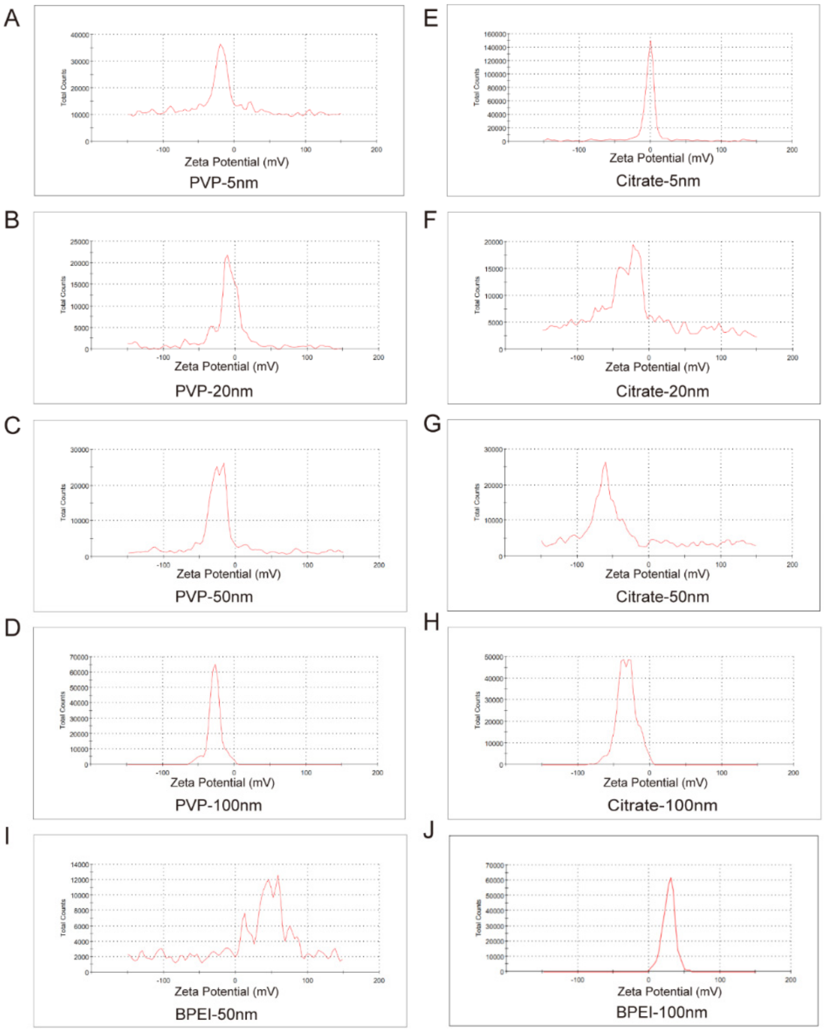

Figure S3. The zeta potential distribution measured via dynamic light scattering. (**A**–**D**) Zeta potential distribution images of PVP modified AgNPs with different particle sizes: 5 nm (**A**), 20 nm (**B**), 50nm (**C**), 100nm (**D**); (**E**–**H**) Zeta potential distribution of citrate-modified AgNPs: 5 nm (**E**), 20 nm (**F**), 50nm (**G**), 100nm (**H**); (**I**,**J**) Zeta potential distribution of BPEI modified AgNPs with different particle sizes: 50nm (**I**), 100nm (**J**).

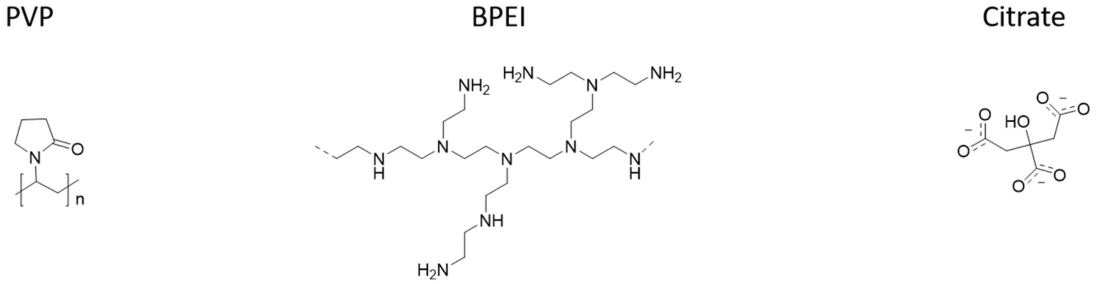

Figure S4. Chemical structure of AgNP surface coatings.

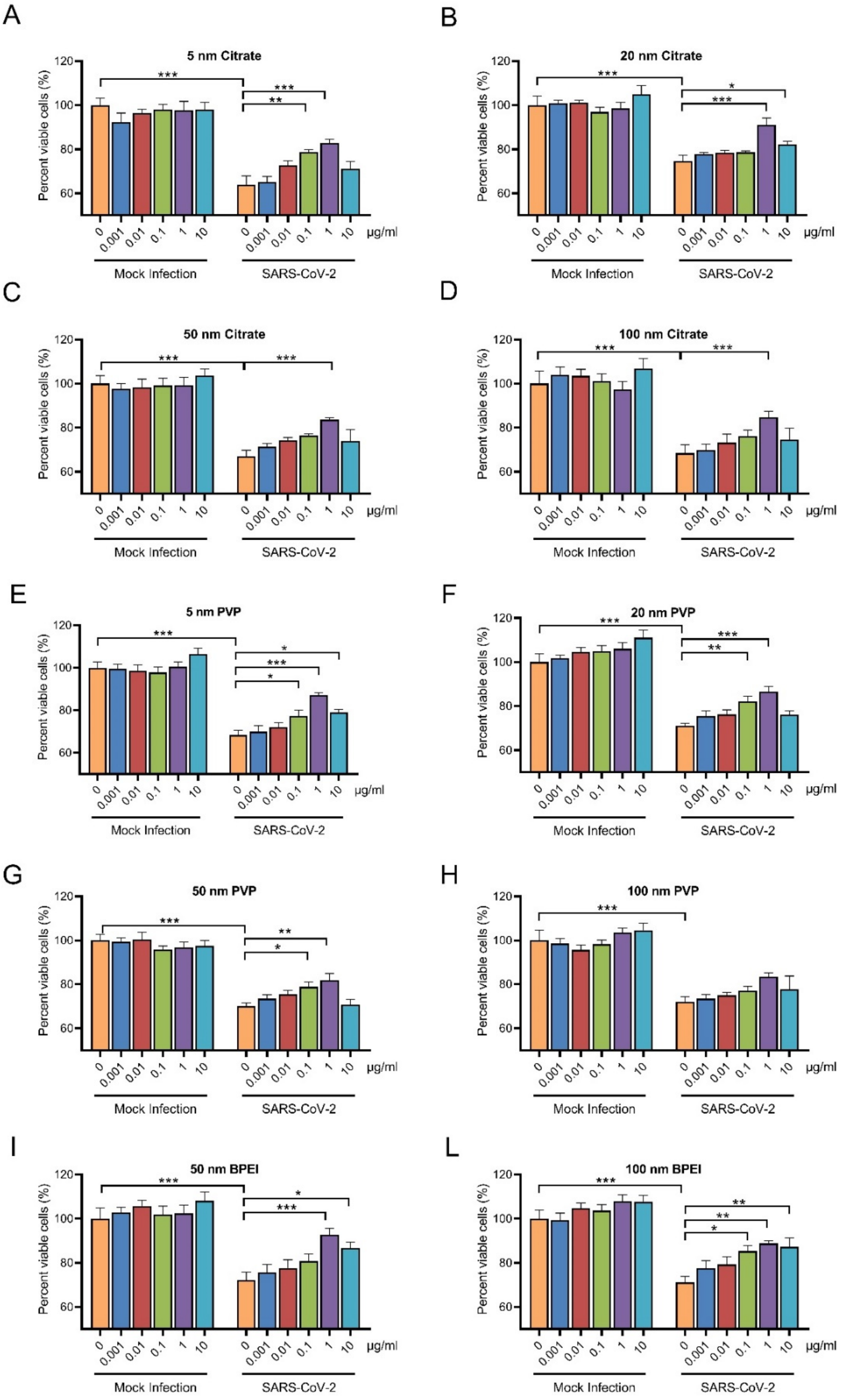

Figure S5. Effects of AgNPs of different concentration on the viability of Vero E6 cells infected with SARS-CoV-2 at 48 h. Vero E6 cells were infected with SARS-CoV-2 (MOI = 0.008). (**A**)5 nm citrate; (**B**)20 nm citrate; (**C**)50 nm citrate; (**D**)100 nm citrate; (**E**)5 nm PVP; (**F**)20 nm PVP; (**G**)50 nm PVP; (**H**)100 nm PVP; (**I**)50 nm BPEI; (**J**)100 nm BPEI. n = 8 groups, * $p < 0.05$, ** $p < 0.01$, *** $p < 0.001$.

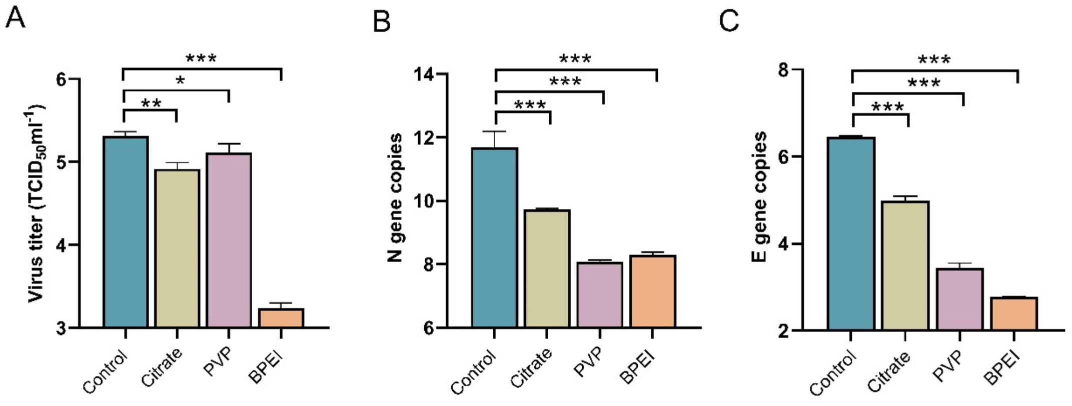

Figure S6. Effect of AgNPs on the replication of SARS-CoV-2 in Vero E6 cells at 48 h. The cells were infected with SARS-CoV-2 (0.008 MOI). Vero E6 cells were treated with citrate, PVP and BPEI coated AgNPs with 50 nm. (**A**) Virus titer; (**B**) Copy number of SARS-CoV-2 N gene; (**C**) Copy number of the SARS-CoV-2 E gene. Three experiments were conducted (n = 3 group), * p <0.05, ** $p < 0.01$, *** $p < 0.001$.
